# *Escherichia coli* ATCC 8739 Adapts to the Presence of Sodium Chloride, Monosodium Glutamate, and Benzoic Acid after Extended Culture

**DOI:** 10.5402/2012/965356

**Published:** 2012-03-05

**Authors:** Chin How Lee, Jack S. H. Oon, Kun Cheng Lee, Maurice H. T. Ling

**Affiliations:** ^1^School of Chemical and Life Sciences, Singapore Polytechnic, Singapore 139651, Singapore; ^2^Department of Zoology, The University of Melbourne, Melbourne, VIC 3010, Australia

## Abstract

*Escherichia coli* is commonly found in intestine of human, and any changes in their adaptation or evolution may affect the human body. The relationship between *E. coli* and food additives is less studied as compared to antibiotics. *E. coli* within our human gut are consistently interacting with the food additives; thus, it is important to investigate this relationship. In this paper, we observed the evolution of *E. coli* cultured in different concentration of food additives (sodium chloride, benzoic acid, and monosodium glutamate), singly or in combination, over 70 passages. Adaptability over time was estimated by generation time and cell density at stationary phase. Polymerase chain reaction (PCR)/restriction fragments length polymorphism (RFLP) using 3 primers and restriction endonucleases, each was used to characterize adaptation/evolution at genomic level. The amplification and digestion profiles were tabulated and analyzed by Nei-Li dissimilarity index. Our results demonstrate that *E. coli* in every treatment had adapted over 465 generations. The types of stress were discovered to be different even though different concentrations of same additives were used. However, RFLP shows a convergence of genetic distances, suggesting the presence of global stress response. In addition, monosodium glutamate may be a nutrient source and support acid resistance in *E. coli*.

## 1. Introduction

A change in the genetic code, also known as mutation, is the primary source of genetic variation which gives rise to diversity within a population. When accumulated over generations, these genetic variations may improve the adaptability; hence, the survival of organisms in different environmental conditions [1, 2]. This may in turn induce or preferentially select for further advantageous changes for better adaptation within the environment [3–5].

Although mutations-conferring advantageous traits have been observed in animals such as lizards [6] and fish [7], it is difficult to study these effects in a laboratory setting due to space and time constraints. For example, it took 36 years for the lizards to show distinct features [6]. On the other hand, bacteria has a number of advantages-fast generation time [2], ability, to fossilize, and resurrection of historical generations [8]. *Escherichia coli*, a common intestinal bacterium, has been used in a long-term evolutionary experiment spanning more than 2 decades [3, 9–11]. A number of stress adaptation studies had demonstrated that the growth phases may impact *E. coli* adaptation. Nair and Finkel [12] suggested that a nonspecific DNA binding protein, dps, may confer multiple stress tolerance at stationary phase, which concur with Jolivet-Gougeon et al. [13]. In addition, the genome of several strains of *E. coli* had been sequenced, representing a reliable source of genetic knowledge.

In terms of the effects of chemical treatments, bacterial resistance and tolerance to antibiotics are well established and the mechanisms have been widely studied [14–17]. In contrast, mechanisms of insusceptibility to nonantibiotic agents, such as food preservatives and antiseptics which might include tolerance or resistance, are less well understood. For example, citric acid inhibits the growth of proteolytic strains of *Clostridium botulinum *[18], sodium chloride can inhibit the growth of many bacteria such as *Listeria monocytogenes* [19], *Ochrobactrum anthropi *[20], and* Lactobacillus plantarum *[21] by lowering the water activity [22], and fatty acid such as formic [23], propionic [14], and acetic acid [24, 25] are also capable of inhibiting bacterial growth.

As an intestinal bacterium, *E. coli* comes into contact with the food and chemicals that we consume. A study which treated pigs with ampicillin, a common antibiotic, demonstrated a significant increase in the occurrence of ampicillin-resistant *E. coli* from 6% to more than 90% after a course of 7 days [26]. It has been suggested that incomplete absorption in the large intestine led to the presence of subtherapeutic doses of antibiotics in the faeces, resulting in evolutionary pressure for intestinal bacteria such as *E. coli* towards antibiotic resistance [27, 28].

Although the interactions between antibiotics and bacteria have been well studied [16, 26, 29, 30], the interactions between food additives and bacteria remain elusive. These food additives may have an impact on the evolution of intestinal flora. It had been demonstrated that benzoic acid [31] and sodium chloride [32, 33] can affect *E. coli* physiology. However, the effect at the genome level is not known.

This paper has two aims. Firstly, we aim to examine the adaptability of *E. coli* ATCC 8739 (a sequenced strain) in a long-term culture environment in the presence of benzoic acid, sodium chloride, and monosodium glutamate (MSG, a common taste enhancer in Asian cooking), singly and in combination. Two concentrations of each additive were used to evaluate the effects of concentration in the adaptability of *E. coli*. Generation time across passages is used as an estimation of adaptation where decreased generation time across passages in an additive demonstrated that the cells are growing faster compared to an earlier passage. Thus, a decrease in generation time across passages indicates that the cells are adapting to the additive, whereas an increase in generation time suggests stress. The rate of decrease is an estimator of the rate of adaptation. Secondly, we aim to estimate the genomic effects of these adaptations using Nei and Li distance to estimate the genetic distances between the samples after polymerase chain reaction (PCR)/restriction fragments length polymorphism (RFLP). 

We hypothesized that, *E. coli* ATCC 8739 is able to adapt to the food additives; thereby, demonstrating decrease in generation time across passages. Generation time analysis demonstrated *E. coli* ATCC 8739 is able to adapt to the additives over extended culture, and DNA fingerprinting suggests that benzoic acid, sodium chloride, and monosodium glutamate are exerting evolutionary pressure on the bacterium.

## 2. Materials and Methods

### 2.1. Main Culture Experiment

Lysophilised *Escherichia coli* ATCC 8739 strain (Reference Passage 4 from ATCC) was revived on nutrient agar plate and incubated at 37°C before inoculating into 8 different treatments supplementation in 10 mL Nutrient Broth. These 8 treatments consist of 4 sets of additives with 2 different concentrations each as shown in Table 1. 

**Figure 1:**
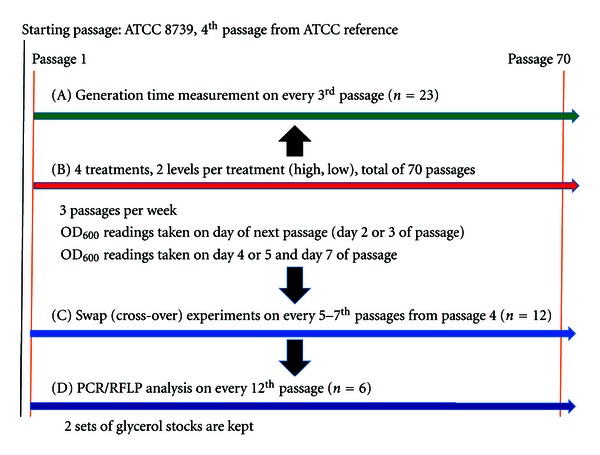
Work-flow of experimental design.

The subculturing and generation time estimation protocol were as follows (Figure 1). Subculturing was performed by transferring 1% (100 *μ*L) of the previous culture on every Monday, Wednesday, and Friday to the next passage in order for adaptation to occur at the stationary phase of growth. Optical density (OD) readings were taken before the next subculture at 600 nm wavelength to estimate the number of generations within the current passage and to also determine the number of cells that are being inoculated into the new passage. Generation time was measured on every 3rd passage. Glycerol stocks for each treatment were made from 1% of the culture for every 12th passage after culturing on MacConkey agar.

### 2.2. Swap Experiment

The swap experiment was done fortnightly (6-7 passages interval), involving the transfer of *Escherichia coli *cells cultured in different treatments to other treatments for the measurement of generation time. Four types of swaps were carried out, whereby the cells were inoculated into the new treatment in a 100 times dilution. The first set of swap involves the inoculation of basal medium- (L SALT) treated cells into the six nonsalt treatments. An example would be inoculating cells grown in L SALT into H MSG treatment. For the second set, cells cultured in high and low concentrations of each treatment were swapped for all treatments. For example, cells growing in H MSG were inoculated into the L MSG media and vice versa. In the third set, cells of high concentration treatments (H MSG, H BA, and H SALT) were each inoculated into the H COMB treatment. The last set is similar to the previous set except that cells of the low concentration were swapped. Cells from low concentration treatments (L MSG, L BA, and L SALT) were each inoculated into L COMB media. OD600 readings were recorded down at intervals, and generation times were calculated for each interval.

### 2.3. Genomic DNA Extraction

Treatment cultures from every 12th passage interval were used for genomic DNA extraction using the phenol-chloroform method of DNA extraction for Gram-negative bacteria [34]. The DNA pellet was air-dried and dissolved to 100 ng/*μ*L in pH 8.0 Tris/HCl buffer and stored at −20°C.

### 2.4. Polymerase Chain Reaction

Each reaction consisted of 50 *μ*L of mixture prepared using 200 ng of DNA template in 10pmoles of DNTPs, 50 pmoles of primer, 1 unit of Taq polymerase, and 1X standard buffer (with 1.5 mM of MgCl_2_) provided by the supplier (New England Biolabs, Inc.). Primer 5, CgCgCTggC; Primer 6, gCTggCggC; Primer 7, CAggCggCg were used separately. The PCR reaction was carried out (Hybaid Limited, PCR express) under the cycling condition of initial denaturation at 95°C for 10 minutes; 35 cycles of amplification at 95°C for 1 minute, 27°C for 1 minute, 72°C for 3 minutes, followed by a final extension at 72°C for 10 minutes before gel electrophoresis in 2% (w/v) agarose gel with 1X GelRed. The primers used were generated by a previously described method [35] using the following rules: (1) the same primer must be suitable as forward and reverse primers, (2) each primer must be between 6 to 15 bases, (3) the predicted amplicon size must be between 300 to 3100 bases in order for resolution in 2% (w/v) agarose gel, and (4) each primer should be predicted to yield 3 or 4 amplicons. 

### 2.5. Restriction Fragments Length Polymorphism

11 *μ*L of PCR product was digested with 1 unit of restriction endonuclease (TaqI, HinfI, or MspI), in a reaction mixture consisting of 1X restriction digestion buffer and 100 ng/*μ*L acetylated BSA made to a total volume of 20 *μ*L with distilled water. HinfI and MspI reaction mixtures were incubated at 37°C, while the TaqI reaction mixture was incubated at 65°C. All reaction mixtures were incubated for 16 hours before analysis in 2% (w/v) agarose gel with 1X GelRed.

### 2.6. Data Analysis

Cell density was calculated from OD600 readings using the correction suggested by Sezonov et al. [36]. Briefly, the cell density is directly proportional to OD600 readings when OD600 reading is below or equal to 0.3, at which the cell density is equivalent to 5 × 10^7^ cells per milliliter. If OD600 reading is above 0.3, the cell density is estimated by the equation of Cell Density = 52137400 × In (OD600 reading) + 118718650. Generation time for all experiments was calculated from difference in cell density at intervals between 120 and 300 minutes after the inoculation of cells into fresh media, and the geometric mean was calculated. Changes in generation time across passages were tested using *t*-test for regression coefficient [37]. The migration distance of the bands of PCR and RFLP of different treatments within the same passage was tabulated and a Nei-Li dissimilarity index (DI) [38], where the maximum value of 1 is obtained when there are no common bands when comparing between the 2 treatments, while a minimum of 0 will be obtained when the 2 treatments have exactly the same bands [35]. The correlation coefficient (CC) value between DIs across passages statistically tested against the CC value of 0.95 (~1) using the *Z*-test for two correlation coefficients [37] where the *P* value of more than 0.05 would indicate that the null hypothesis (CC is equal to 0.95) is not rejected.

## 3. Results

### 3.1. Generation Time

Analysis of the generation times showed that all eight treatments over the passages displayed different rates of decreasing generation times as shown in Table 2. The steepest decline in generation time occurs in H COMB treatment where approximately 2.02 minutes reduction in generation time per passage over 70 passages was observed, followed by L MSG (1.87 minutes), L BA (1.39 minutes), L SALT (1.24 minutes), L COMB (1.22 minutes), H BA (1.15 minutes), H SALT (1.12 minutes), and finally H MSG (0.906 minutes). The regression intercept may be used to estimate the generation time of the cells in each treatment media for the first passage which is indicative of the level of initial stress on the cells. On this basis, the treatment exerting the highest level of stress on the cells would be H COMB, followed by L MSG, H BA, L BA, L COMB, L SALT, H SALT, and H MSG.

### 3.2. Swap Experiment

The linear regression of the generation time across passages demonstrated that the gradients of the equations are not equal to zero which indicates that the generation times are not constant for the six swaps. Although there are changes in the general trend of generation time across the passages, the *P* values calculated for the six swaps were more than 0.05 which is not significant: L SALT cells to H MSG media, 0.475509; L SALT to L MSG media is 0.421721; L SALT cells to H BA media is 0.250415, L SALT cells to L BA media is 0.4660235; L SALT cells to H COMB media is 0.484887; L SALT cells to L COMB is 0.443381. 

The generation times trend of the four swaps (MSG, BA, Salt, and Combination) from low-treatment to high-treatment over 12 swaps change in a decreasing manner (Table 3). With reference to the regression equations, the linear regression gradient of low treatment cells into high treatment media for combination treatment is the steepest followed by that for BA, MSG, and salt treatments. At swap count between eight and nine, the generation time is almost the same for MSG, salt, and combination, but the generation time for BA is still distantly higher.

The generation time trends of the four swaps (MSG, BA, salt, and combination), from high treatment to low treatment over 12 swaps, changed in a decreasing manner. With reference to the regression equations, the linear regression gradient of high-treatment cells into low-treatment media for MSG treatment was the steepest followed by that for BA, combination, and salt treatments. The generation time of high-treatment cells into low-treatment media for MSG treatment was almost the same as for salt treatment between swap count four and five, and as for combination treatment between swap count eight and nine. The generation time for BA is consistently-lowest among the treatments.

The generation time of swapping high-concentration treated cells into high-combination medium showed similar trends. At the 8th swap, H BA suddenly increased in generation time, due to the retarding growth. The OD600 reading ranges from 0.069 to 0.084 over a period of three days. After the 10th swap, generation time for all treatments remain, constant at 200 minutes till the 12th swap.

The generation time of swapping low-concentration treated cells in to low-combination medium showed similar trends. At the 2nd swap, L Salt suddenly increased in generation time, due to the slow growth. The OD600 reading ranges from 0.077 to 0.545 over a period of 3 days. After the 4th swap, generation time for all treatment followed a similar trend till the 12th swap.

### 3.3. PCR/RFLP

Electrophoresis agarose gels of the PCR and RFLP products for the eight treatments were used to study the differences between the genome of the *E. coli* cells of the treatments across the passages. Nei-Li dissimilarity index (DI), which had been shown to be suitable for RFLP [39], was utilised to mathematically calculate the dissimilarity between pair-wise comparisons of the treatments.

**Figure 2:**
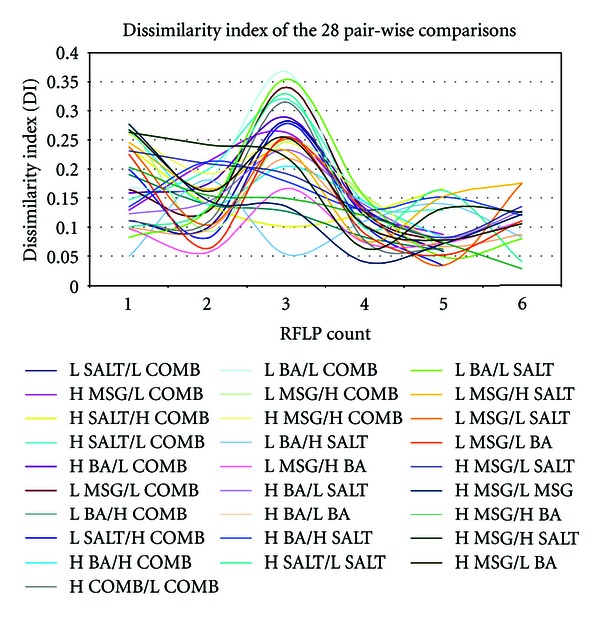
Dissimilarity index of the 28 pair-wise comparisons for the 6 PCR/RFLP. The data points of comparisons with H COMB or L COMB of PCR/RFLP number 6 were excluded from this and subsequent analysis due to unusually high dissimilarity index which is caused by an error in the PCR of H COMB and L COMB of PCR/RFLP number 6.

The dissimilarity index of the 28 comparisons showed a trend of convergence from PCR/RFLP number 4 onwards (Figure 2).

**Figure 3:**
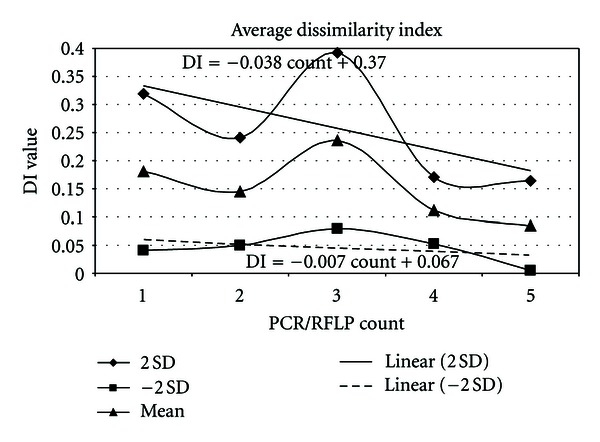
Estimation of the DI for each PCR/RFLP count. The mean and ±2 SD (standard deviations) were calculated from Nei and Li distances of the 28 pair-wise comparisons. The linear regression lines were calculated from ±2 SD values in order to indicate whether the genetic distances are converging or diverging.

This trend is further elaborated with the estimation of the maximum and minimum mean values (Figure 3) which shows converging linear regression line across the 6 PCR/RFLP.

Six resulting effects (Table 4) obtained from the comparisons were analysed. The similarity among the six resulting effects is that each type of effects had two originating comparisons. Therefore by plotting the two comparisons against each other and testing for significance, we can deduce whether the genomic differences in each of the two comparisons are actually a consequent effect from the resulting effects.

All resulting effects were not statistically significant except for 10BA + SALT. This suggests that the PCR/RFLP-inferred genetic distance between H MSG and H COMB, and H BA and H SALT varied independently (not correlated).

## 4. Discussion 

In this paper, we present one of the first comprehensive investigations of the effect of *E. coli* cells' adaptations to a variety of food additives using a long-term culture approach. Our results suggest that cells grown under different stress condition are able to adapt to the environment which can be observed by decreased generation time and genetic variations. 

### 4.1. Nutrient Broth Does Not Prime Cells for Growth in Other Treatments

Since *E. coli* cells were grown in NB with the various supplementations of treatments, it was important that any changes to the cells were a direct result of the treatments rather than from the NB. The generation time trend for *E. coli* cells from L SALT (NB) inoculated into six different media (Table 3) showed that nutrient broth did not appear to impact on adaptability as none of the regression gradients were statistically different from a gradient of zero suggesting that the general generation time trend remained almost the same; therefore, nutrient broth (L SALT media) was unlikely the cause of any adaptations observed. 

### 4.2. Cells Adapt to Their Individual Treatments

The low-concentration treated cells were observed to be adapting to their environment as seen from the decreasing generation time. The cells are dividing at a faster rate with increasing passages suggesting lowered stress level in later passages comparing to early passages (Table 3). However, as the concentration of additives is the same throughout the passages, decreased generation time suggests that the cells are adapting to the stress. Low-treatment cells inoculated into high-treatment media also showed decrease in generation time across the swaps. Growth rate has been used as a measure for adaptation to a stressed environment in previous studies where Chen and Shakhnovich [40] had demonstrated increase in growth rate of 35 bacterial species upon adaptation to thermal stress. In addition, Zhu and Yang [41] also demonstrated increase in growth rate; thus, decrease in generation time, when *Clostridium tyrobutyricum* adapts to the presence of butyric acid. Our results suggest that the low-treatment cells had gradually adapted to its own individual treatment before the swap, causing it to be less stressed when swapped into high-treatment media. 

The generation times of cells from low single treatments to L COMB were observed to increase gradually across passages (Table 3). This suggests that low MSG, BA, or Salt treatments were not stressful enough to induce significant adaptations such that when they were placed into the L COMB treatment which now contains additional stress inducers, the cells could not cope. This may suggest that the cells may be gradually optimized to grow in a specific treatment; thus, increasingly specialized to their specific environment. Similar cases had been reported in other evolutionary studies using *E. coli* [42–44]. The effects of L COMB did not increase the adaptability but instead decreases it as seen from the generation time analysis (Table 2). This suggests that L COMB may be less stressful compared to the individual treatments. It has been suggested that the presence of MSG counteracts the effects of drop in pH caused by BA [14]. This is achieved by increasing the resistance of *E. coli* cells against the lowered pH, which will otherwise kill the cells. This suggests that the effects of L MSG and L BA cancel each other out, leaving only L SALT which is further supported by the similarity between the adaptability of L SALT (−1.24 minutes per generation, Table 2) and L COMB (−1.22 minutes per generation, Table 2). 

*E. coli* cultured in H COMB treatment had the greatest decline in the generation time over 70 passages. Since higher stress level may force the cells to adapt quickly in order to survive [45, 46], suggesting that the *E. coli* cells in H COMB treatment experienced the highest level of stress among the eight treatments (Table 2) in contrast to H MSG which induced the lowest decrease in generation time. This may suggest that the presence of glutamate in MSG may be aiding the growth of cells [47] as glutamate can serve as an additional source of nutrient for the cells. Thus, H MSG may cause the least amount of stress but instead led to better growth resulting in a lower rate of adaptation; hence, the lowest decrease in generation time. 

When high-concentration treated cells were swapped into low-treatment media containing the same type of stress, reduced generation time was observed. This suggests that these cells have adapted to its individual treatment. Although both high and low concentrations appear to result in some adaptations as measured by generation time, the rate of adaptation differs. The general decline in generation times of the cells from low-treatment to high-treatment media is steeper than that of the reverse. High-concentration treated cells inoculated into low-concentration treatment media appeared more stressed. This is surprising as low-concentration treated cells inoculated into high-treatment media should experience more stress than high-concentration treated cells inoculated into low-treatment media [48], provided that the type of stress is similar. A possible explanation for this is that the type of stress may differ, even though low and high treatment contained the same type of additives and differing only in concentration. This may be explained by the catabolism of stress-induced molecules [49]. In Takatsu [49], the level of cardiolipin, a salt stress marker of *Staphylococcus aureus*, took longer to return to basal level upon reculture in basal media after stressed in 5% NaCl than in 10% NaCl. This may suggest that function of stress-induced molecules may be concentration dependent. It may be plausible that high-concentration treated cells may induce stress-induced molecules which may add to the level of metabolic stress at a lower concentration. However, the adaptive nature of different concentrations requires further studies. 

The swap from individual high-concentration treatments (H MSG, H BA, and H SALT) to H COMB showed decreased generation time. This may suggest that pretreatment of cells in a stressed environment may condition them to adapt to another stress environment which has been demonstrated by other adaptation studies using *E. coli* [50–53]. However, the swap from individual low-concentration treatments (L MSG, L BA, and L SALT) to L COMB showed the opposite trend with increased generation time. This further corroborates that the adaptive nature of different concentrations may be different; thus, requires further studies. 

### 4.3. Cells from Different Treatments Become Genetically Similar

Our results from PCR/RFLP showed a converging trend in DI indicating that the *E. coli* from all treatments are getting similar (Figure 3) suggesting that they mutate in a similar manner. This suggests that they may evolve the same type of stress mechanism and DNA repair. However, all the treatments originate from the same bacterial clone, suggesting that the initial stress adaptation may involve mutation as it has been suggested that hypermutation is a feature in initial stress adaptations [54]. 

It is known that *E. coli* exposed to stresses would respond to counteract the effects. Tucker et al. [55] has shown that *E. coli* in nitric oxide (NO) will reduce the NO to nitrous oxide under anaerobic conditions which is harmless to the cell. In another study, *E. coli* produces methylglyoxal to counteract toxic electrophiles [56]. 

While the cells from all treatments may have experienced different types and levels of stresses, it is likely that the cells might have adapted and activated similar stress-responsive mechanism by evolving similarly. This interpretation is supported by a number of studies suggesting the presence of global stress response in *E. coli* [57–59]. In addition, Cebrián et al. [50] found that adaptation to pH stress may protect *Staphylococcus aureus* against oxidative stress by hydrogen peroxide, suggesting that adaptation to particular stress may confer tolerance to other stresses. 

In this paper, the similar response and mutations to the *E. coli* of the treatments could not be determined for the whole genome. Primers 5, 6, and 7 amplified a random sample amounting to 0.37% of the whole genome which is a limitation of this study. However, several studies had demonstrated that PCR-based DNA fingerprinting using a small number of primers is suitable to examine genetic diversity in* E. coli* [60], *Candida dubliniensis* [61], and mackerel [62]. Another limitation is that DNA fingerprinting was performed on the entire population and not on isolated colonies. Hence, this paper can only imply on areas of the genome that were amplified, and analysis was approaching genetic similarity at a population scale. This method had been used other studies examining metagenomics in environmental bacterial samples [63, 64] and human myopia [65]. The genes responsible for stress-handling mechanism may also not be present in the amplified regions of the genome. 

It is unlikely that the genetic distance of *E. coli* in the eight treatments reaches zero as a population, suggesting that the declining trend is likely to taper off. In addition, spontaneous mutation may prohibit them from being identical. On the other hand, previous studies in *E. coli* [44] and *Herminiimonas arsenicoxydans* [66] demonstrated the presence of ecological specialization. Our results showed that the generation time decreased over passages, suggesting the possibility of ecological specialization. This may indicate the presence of both global stress response and ecological specialization in *E. coli*. Global stress response allows for adaptation to new stress environments, but extended stress may lead to ecological specialization. Hence, it may be hypothesized in future studies that continued culture may lead to ecological specialization which may be seen as a divergence in the genetic distance. 

Statistical analysis of the selected comparisons indicates that the effects of all the treatments were insignificant except 10BA + SALT (Table 4). Statistical tests for MSG and BA effect suggested that different gels provided constant results; thus, suggesting reliability in our study. 

Statistical tests suggest that MSG and BA, and MSG and SALT are likely to interact with each other (Table 4). However, 10BA + SALT do not appear to have an interacting effect. This suggests that high-combination media contains 10MSG + 10BA + SALT and the interacting effects of MSG and BA, and MSG and salt. Results from swap analysis indicated that low-salt cells when swapped to high-combination media showed an increase in generation time. This suggests that the high stress in high combination media results in difficulties for the cells from L SALT to grow and caused an increase in doubling time. This might be due to the additional combined stress produced by MSG interacting with BA and SALT. The presence of the additional interacting stress of the combination treatment can also be observed from the analysis of generation time where the stress level of H COMB is much higher than the three individual high-concentration treatments. 

However, some interacting effects may not be hindering the growth of the bacteria. BA kills bacteria by lowering the pH of the media, whereas MSG has the effects of pH resistance on the cells. Hence, the presence of MSG may aid the growth of the *E. coli* living in low-pH environment [14] caused by the presence of BA. On the other hand, combined effects from MSG + S could be harmful to the cells as salt may increase the high sodium content provided by MSG in media. The high-sodium environment actually changes the environment to be even more selective.

## 5. Conclusion and Future Work 

This paper had demonstrated that *E. coli* is able to adapt to food additives over an extended period of time by observing the decreased in generation time over a period of 70 passages. This may have implications in using sublethal doses of bacteriocidal agents such as disinfectants and preservatives. Our results suggest the likelihood of both global stress responses and ecological specialization. Hence, it may be hypothesized that increasing passages may demonstrate a shift towards ecological specialization. 

## Figures and Tables

**Table 1:** Additives for 8 treatments. With the exception of sodium chloride (SALT), high-concentration treatment contains 10 times the additive compared to the corresponding low-concentration treatment.

Set	Treatment	Additives
Monosodium glutamate (MSG)	High MSG (H MSG)	0.025% (w/v) MSG
	Low MSG (L MSG)	0.0025% (w/v) MSG

Benzoic acid (BA)	High BA (H BA)	0.025% (w/v) BA
	Low BA (L BA)	0.0025% (w/v) BA

Sodium chloride (SALT)	High SALT (H SALT)	1% (w/v) NaCl
	Low SALT (L SALT)	No additive

Combination (COMB)	High COMB (H COMB)	0.025% (w/v) MSG, 0.025% (w/v) BA, and 1% (w/v) NaCl
	Low COMB (L COMB)	0.0025% (w/v) MSG and 0.0025% (w/v) BA

**Table 2:** Linear regression equations of generation times of the eight treatments. Negative linear regression gradient represents a decrease in generation time as the passage number increases. For example, a gradient of −2.02 in H COMB represents that the generation time decreases by 2.02 minutes in each passage. The intercept can be used as an estimator of the generation time at Passage 1.

Treatment	Linear regression equation
H COMB	Generation time = −2.02 Passage number + 271
L MSG	Generation time = −1.87 Passage number + 225
L BA	Generation time = −1.39 Passage number + 196
L SALT	Generation time = −1.24 Passage number + 189
L COMB	Generation time = −1.22 Passage number + 190
H BA	Generation time = −1.15 Passage number + 203
H SALT	Generation time = −1.12 Passage number + 181
H MSG	Generation time = −0.91 Passage number + 171

**Table 3:** Linear regression equations of generation times of the swap treatments. The linear regression gradient represents the change of generation time between each swap counts (5–7 passages).

Original treatment	New treatment	Linear regression equation
	High MSG	Generation time = 0.90 Swap count + 151
	Low MSG	Generation time = −5.87 Swap count + 230
Low SALT	High BA	Generation time = −6.68 Swap count + 385
	Low BA	Generation time = 1.29 Swap count + 151
	High COMB	Generation time = 2.34 Swap count + 312
	Low COMB	Generation time = 3.44 Swap count + 138

Low MSG	High MSG	Generation time = −4.37 Swap count + 237
Low BA	High BA	Generation time = −15.02 Swap count + 398
Low SALT	High SALT	Generation time = −10.67 Swap count + 290
Low COMB	High COMB	Generation time = −17.18 Swap count + 348

High MSG	Low MSG	Generation time = −6.91 Swap count + 214
High BA	Low BA	Generation time = −5.25 Swap count + 182
High SALT	Low SALT	Generation time = −1.53 Swap count + 193
High COMB	Low COMB	Generation time = −4.20 Swap count + 192

High MSG		Generation time = −63.58 Swap count + 814
High BA	High COMB	Generation time = −37.07 Swap count + 849
High SALT		Generation time = −29.26 Swap count + 480

Low MSG		Generation time = 4.55 Swap count + 164
Low BA	Low COMB	Generation time = 14.77 Swap count + 109
Low SALT		Generation time = 3.44 Swap count + 138

**Table 4:** Tabulation of *P* value for the resulting effects. As the additive concentration of the high treatment is 10 times that of low treatment for BA and MSG, BA refers to L BA additive, while 10BA refers to H BA additive. The resulting effects represent the difference in the treatments. For example, MSG is the difference between L MSG and L SALT.

PCR-RFLP comparison	Resulting effects	Correlation coefficient	*Z* Statistic	*P* value	Significant
L MSG/L SALT, L BA/L COMB	MSG	0.786	−0.944	0.173	No
L MSG/L COMB, L BA/L SALT	BA	0.934	−0.175	0.431	No
L MSG/L BA, L SALT/L COMB	BA + MSG	0.764	−1.012	0.156	No
H MSG/H SALT, H BA/H COMB	10MSG + SALT	0.631	−1.333	0.091	No
H MSG/H COMB, H BA/H SALT	10BA + SALT	0.142	−2.068	0.019	Yes
H MSG/H BA, H SALT/H COMB	10MSG + 10BA	0.437	−0.167	0.434	No

## References

[1] Lenski RE, Rose MR, Simpson SC, Tadler SC (1991). Long-term experimental evolution in *Escherichia coli*—I. Adaptation and divergence during 2000 generations. *American Naturalist*.

[2] Travisano M (1997). Long-term experimental evolution in *Escherichia coli*—VI. Environmental constraints on adaptation and divergence. *Genetics*.

[3] Blount ZD, Borland CZ, Lenski RE (2008). Historical contingency and the evolution of a key innovation in an experimental population of *Escherichia coli*. *Proceedings of the National Academy of Sciences of the United States of America*.

[4] Hurst LD (2009). Fundamental concepts in genetics: genetics and the understanding of selection. *Nature Reviews Genetics*.

[5] Philippe N, Pelosi L, Lenski RE, Schneider D (2009). Evolution of penicillin-binding protein 2 concentration and cell shape during a long-term experiment with *Escherichia coli*. *Journal of Bacteriology*.

[6] Herrel A, Huyghe K, Vanhooydonck B (2008). Rapid large-scale evolutionary divergence in morphology and performance associated with exploitation of a different dietary resource. *Proceedings of the National Academy of Sciences of the United States of America*.

[7] Herrel A, Choi HF, Dumont E (2011). Burrowing and subsurface locomotion in anguilliform fish: behavioral specializations and mechanical constraints. *Journal of Experimental Biology*.

[8] Achá SJ, Kühn I, Mbazima G, Colque-Navarro P, Möllby R (2005). Changes of viability and composition of the *Escherichia coli* flora in faecal samples during long time storage. *Journal of Microbiological Methods*.

[9] Lenski RE (1988). Experimental studies of pleiotropy and epistasis in *Escherichia coli*—I. Variation in competitive fitness among mutants resistant to virus T4. *Evolution*.

[10] Travisano M, Lenski RE (1996). Long-term experimental evolution in *Escherichia coli*. IV. Targets of selection and the specificity of adaptation. *Genetics*.

[11] Woods RJ, Barrick JE, Cooper TF, Shrestha U, Kauth MR, Lenski RE (2011). Second-order selection for evolvability in a large *Escherichia coli* population. *Science*.

[12] Nair S, Finkel SE (2004). Dps protects cells against multiple stresses during stationary phase. *Journal of Bacteriology*.

[13] Jolivet-Gougeon A, David-Jobert S, Tamanai-Shacoori Z, Ménard C, Cormier M (2000). Osmotic stress-induced genetic rearrangements in *Escherichia coli* H10407 detected by randomly amplified polymorphic DNA analysis. *Applied and Environmental Microbiology*.

[14] Bhagwat AA, Chan L, Han R (2005). Characterization of enterohemorrhagic *Escherichia coli* strains based on acid resistance phenotypes. *Infection and Immunity*.

[15] De Groote VN, Fauvart M, Kint CI (2011). *Pseudomonas aeruginosa* fosfomycin resistance mechanisms affect non-inherited fluoroquinolone tolerance. *Journal of Medical Microbiology*.

[16] Kim JS, Heo P, Yang TJ (2011). Bacterial persisters tolerate antibiotics by not producing hydroxyl radicals. *Biochemical and Biophysical Research Communications*.

[17] Martinez M, Silley P (2010). Antimicrobial drug resistance. *Handbook of Experimental Pharmacology*.

[18] Russell AD (1991). Mechanisms of bacterial resistance to non-antibiotics: food additives and food and pharmaceutical preservatives. *Journal of Applied Bacteriology*.

[19] Garner MR, James KE, Callahan MC, Wiedmann M, Boor KJ (2006). Exposure to salt and organic acids increases the ability of *Listeria monocytogenes* to invade Caco-2 cells but decreases its ability to survive gastric stress. *Applied and Environmental Microbiology*.

[20] Kesserü P, Kiss I, Bihari Z, Polyák B (2002). The effects of NaCl and some heavy metals on the denitrification activity of *Ochrobactrum anthropi*. *Journal of Basic Microbiology*.

[21] Glaasker E, Tjan FSB, Ter Steeg PF, Konings WN, Poolman B (1998). Physiological response of *Lactobacillus plantarum* to salt and nonelectrolyte stress. *Journal of Bacteriology*.

[22] Verluyten J, Messens W, De Vuyst L (2004). Sodium chloride reduces production of Curvacin A, a bacteriocin produced by *Lactobacillus curvatus* strain LTH 1174, originating from fermented sausage. *Applied and Environmental Microbiology*.

[23] Dashper SG, Reynolds EG (2000). Effects of organic acid anions on growth, glycolysis, and intracellular pH of oral Streptococci. *Journal of Dental Research*.

[24] Kanchanarach W, Theeragool G, Inoue T, Yakushi T, Adachi O, Matsushita K (2010). Acetic acid fermentation of *Acetobacter pasteurianus*: relationship between acetic acid resistance and pellicle polysaccharide formation. *Bioscience, Biotechnology and Biochemistry*.

[25] Roe AJ, O’Byrne C, McLaggan D, Booth IR (2002). Inhibition of *Escherichia coli* growth by acetic acid: a problem with methionine biosynthesis and homocysteine toxicity. *Microbiology*.

[26] Bibbal D, Dupouy V, Prere MF, Toutain PL, Bousquet-Melou A (2009). Relatedness of *Escherichia coli* strains with different susceptibility phenotypes isolated from swine feces during ampicillin treatment. *Applied and Environmental Microbiology*.

[27] Bibbal D, Dupouy V, Ferré JP (2007). Impact of three ampicillin dosage regimens on selection of ampicillin resistance in Enterobacteriaceae and excretion of blaTEM genes in swine feces. *Applied and Environmental Microbiology*.

[28] Furtula V, Farrell EG, Diarrassouba F, Rempel H, Pritchard J, Diarra MS (2010). Veterinary pharmaceuticals and antibiotic resistance of *Escherichia coli* isolates in poultry litter from commercial farms and controlled feeding trials. *Poultry Science*.

[29] Soufi L, Sáenz Y, Vinué L (2011). *Escherichia coli* of poultry food origin as reservoir of sulphonamide resistance genes and integrons. *International Journal of Food Microbiology*.

[30] Costa D, Poeta P, Sáenz Y (2008). Mechanisms of antibiotic resistance in *Escherichia coli* isolates recovered from wild animals. *Microbial Drug Resistance*.

[31] Lambert LA, Abshire K, Blankenhorn D, Slonczewski JL (1997). Proteins induced in *Escherichia coli* by benzoic acid. *Journal of Bacteriology*.

[32] Casey PG, Condon S (2002). Sodium chloride decreases the bacteriocidal effect of acid pH on *Escherichia coli* O157 : H45. *International Journal of Food Microbiology*.

[33] Hajmeer M, Ceylan E, Marsden JL, Fung DYC (2006). Impact of sodium chloride on *Escherichia coli* O157 : H7 and Staphylococcus aureus analysed using transmission electron microscopy. *Food Microbiology*.

[34] Cheng HR, Jiang N (2006). Extremely rapid extraction of DNA from bacteria and yeasts. *Biotechnology Letters*.

[35] Lee CH, Oon JSH, Lee KC, Ling MH (2010). Bactome—I. Python in DNA fingerprinting. *The Python Papers*.

[36] Sezonov G, Joseleau-Petit D, D’Ari R (2007). *Escherichia coli* physiology in Luria-Bertani broth. *Journal of Bacteriology*.

[37] Gopal KK (2006). *100 Statistical Tests*.

[38] Nei M, Li WH (1979). Mathematical model for studying genetic variation in terms of restriction endonucleases. *Proceedings of the National Academy of Sciences of the United States of America*.

[39] Chay ZE, Lee CH, Oon JSH, Lee KC, Ling MH (2010). Russel and Rao coefficient is a suitable substitute for dice coefficient in studying restriction mapped genetic distances of *Escherichia coli*. *Computational and Mathematical Biology*.

[40] Chen P, Shakhnovich EI (2010). Thermal adaptation of viruses and bacteria. *Biophysical Journal*.

[41] Zhu Y, Yang ST (2003). Adaptation of *Clostridium tyrobutyricum* for enhanced tolerance to butyric acid in a fibrous-bed bioreactor. *Biotechnology Progress*.

[42] Dykhuizen DE, Dean AM (2004). Evolution of specialists in an experimental microcosm. *Genetics*.

[43] Zhong S, Khodursky A, Dykhuizen DE, Dean AM (2004). Evolutionary genomics of ecological specialization. *Proceedings of the National Academy of Sciences of the United States of America*.

[44] Zhong S, Miller SP, Dykhuizen DE, Dean AM (2009). Transcription, translation, and the evolution of specialists and generalists. *Molecular Biology and Evolution*.

[45] de Paepe M, Gaboriau-Routhiau V, Rainteau D, Rakotobe S, Taddei F, Cerf-Bensussan N (2011). Trade-Off between bile resistance and nutritional competence drives *Escherichia coli* diversification in the mouse gut. *PLoS Genetics*.

[46] Ferenci T, Spira B (2007). Variation in stress responses within a bacterial species and the indirect costs of stress resistance. *Annals of the New York Academy of Sciences*.

[47] Walther D, Strassburg K, Durek P, Kopka J (2010). Metabolic pathway relationships revealed by an integrative analysis of the transcriptional and metabolic temperature stress-response dynamics in yeast. *Omics*.

[48] Doudoroff M (1940). Experiments on the adaptation of *Escherichia coli* to sodium chloride. *The Journal of General Physiology*.

[49] Takatsu T (1975). Adaptive changes in cardiolipin content of staphylococcus aureus grown in different salt concentrations. *Acta Medica Okayama*.

[50] Cebrián G, Sagarzazu N, Pagán R, Condón S, Mañas P (2010). Development of stress resistance in *Staphylococcus aureus* after exposure to sublethal environmental conditions. *International Journal of Food Microbiology*.

[51] Oulkheir S, Ounine K, Elhaloui NE (2007). The effect of salt concentration and pH on the heat resistance of *Escherichia coli* in typtic soy both. *Acta Microbiologica et Immunologica Hungarica*.

[52] Tosun H, Gönül SA (2005). The effect of acid adaptation conditions on heat resistance of *Escherichia coli* O157: H7. *Polish Journal of Microbiology*.

[53] Wiegand KM, Ingham SC, Ingham BH (2009). Survival of *Escherichia coli* 0157:h7 in ground beef after sublethal heat shock and subsequent isothermal cooking. *Journal of Food Protection*.

[54] Jayaraman R (2011). Hypermutation and stress adaptation in bacteria. *Journal of Genetics*.

[55] Tucker NP, D'autréaux B, Spiro S, Dixon R (2006). Mechanism of transcriptional regulation by the Escherichia coli nitric oxide sensor NorR. *Biochemical Society Transactions*.

[56] Ferguson GP (1999). Protective mechanisms against toxic electrophiles in *Escherichia coli*. *Trends in Microbiology*.

[57] Bury-Moné S, Nomane Y, Reymond N (2009). Global analysis of extracytoplasmic stress signaling in *Escherichia coli*. *PLoS Genetics*.

[58] Cuny C, Lesbats M, Dukan S (2007). Induction of a global stress response during the first step of *Escherichia coli* plate growth. *Applied and Environmental Microbiology*.

[59] Gou N, Onnis-Hayden A, Gu AZ (2010). Mechanistic toxicity assessment of nanomaterials by whole-cell-array stress genes expression analysis. *Environmental Science and Technology*.

[60] Ouyang-Latimer J, Ajami NJ, Jiang ZD (2010). Biochemical and genetic diversity of enterotoxigenic *Escherichia coli* associated with diarrhea in United States students in cuernavaca and guadalajara, Mexico, 2004-2007. *Journal of Infectious Diseases*.

[61] Jewtuchowicz VM, Mujica MT, Malzone MC (2009). Genetic relatedness of subgingival and buccal *Candida dubliniensis* isolates in immunocompetent subjects assessed by RAPD-PCR. *Journal of Oral Microbiology*.

[62] Darlina MN, Masazurah AR, Jayasankar P, Jamsari AFJ, Siti AMN (2011). Morphometric and molecular analysis of mackerel (Rastrelliger spp) from the west coast of Peninsular Malaysia. *Genetics and Molecular Research*.

[63] Chen Y, Dumont MG, Neufeld JD (2008). Revealing the uncultivated majority: combining DNA stable-isotope probing, multiple displacement amplification and metagenomic analyses of uncultivated Methylocystis in acidic peatlands. *Environmental Microbiology*.

[64] Ramond J-B, Petit F, Quillet L, Ouddane B, Berthe T (2011). Evidence of methylmercury production and modification of the microbial community structure in estuary sediments contaminated with wastewater treatment plant effluents. *Marine Pollution Bulletin*.

[65] Yip SP, Leung KH, Fung WY, Ng PW, Sham PC, Yap MKH (2011). A DNA pooling-based case-control study of myopia candidate genes COL11A1, COL18A1, FBN1, and PLOD1 in a Chinese population. *Molecular Vision*.

[66] Cleiss-Arnold J, Koechler S, Proux C (2010). Temporal transcriptomic response during arsenic stress inHerminiimonas arsenicoxydans. *BMC Genomics*.

